# Discovering the Biological Target of 5-*epi*-Sinuleptolide Using a Combination of Proteomic Approaches

**DOI:** 10.3390/md15100312

**Published:** 2017-10-13

**Authors:** Elva Morretta, Roberta Esposito, Carmen Festa, Raffaele Riccio, Agostino Casapullo, Maria Chiara Monti

**Affiliations:** 1 Department of Pharmacy, University of Salerno, Via Giovanni Paolo II 132, 84084 Fisciano, Salerno, Italy; emorretta@unisa.it (E.M.); roesposito@unisa.it (R.E.); casapullo@unisa.it (A.C.); 2 PhD Program in Drug Discovery and Development; University of Salerno, Via Giovanni Paolo II 132, 84084 Fisciano, Salerno, Italy; 3 Department of Pharmacy, University of Naples “Federico II”, 80131 Naples, Italy; carmen.festa@unina.it

**Keywords:** marine bioactive compounds, target identification, affinity chromatography, DARTS (drug affinity responsive target stability)

## Abstract

Sinuleptolide and its congeners are diterpenes with a norcembranoid skeleton isolated from the soft coral genus *Sinularia*. These marine metabolites are endowed with relevant biological activities, mainly associated with cancer development. 5-*epi*-sinuleptolide has been selected as a candidate for target discovery studies through the application of complementary proteomic approaches. Specifically, a combination of conventional chemical proteomics based on affinity chromatography, coupled with high-resolution mass spectrometry and bioinformatics, as well as drug affinity responsive target stability (DARTS), led to a clear identification of actins as main targets for 5-*epi*-sinuleptolide. Subsequent in-cell assays, performed with cytochalasin D as reference compound, gave information on the ability of 5-*epi*-sinuleptolide to disrupt the actin cytoskeleton by loss of actin fibers and formation of F-actin amorphous aggregates. These results suggest the potential application of 5-*epi*-sinuleptolide as a useful tool in the study of the molecular processes impaired in several disorders in which actin is thought to play an essential role.

## 1. Introduction

**Figure 1:**
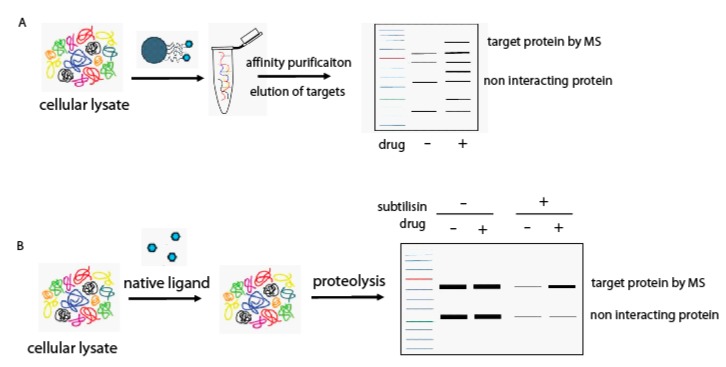
(**A**) In vitro chemical proteomics workflow based on affinity purification followed by nano-liquid chromatography (LC) tandem mass spectrometry (MS/MS) for targets identifications; (**B**) DARTS workflow followed by Western blotting analysis.

Marine environments account for around half of the world’s biodiversity and are a huge resource of structurally and biologically relevant compounds [1]. Most natural marine products are produced by invertebrates, such as tunicates, mollusks, soft corals, sponges and bryozoans, and are currently undergoing advanced preclinical and clinical evaluations [2]. Among soft corals, the genus *Sinularia* represents a leading portion of the biomass in tropical reefs, and is well known to produce a rich collection of secondary metabolites, such as sesquiterpenes, diterpenes, polyhydroxylated steroids, and polyamines, providing a vast spectrum of biological activity [3,4,5]. One of the most interesting metabolites isolated by a *Sinularia* species is sinuleptolide, a norcembranoid with interesting biological functions, such as anticancer and anti-virus activities. Sinuleptolide is able to modulate in vitro lipopolysaccharide-induced TNF-α and nitric oxide production [6], and is cytotoxic against human oral epidermoid carcinoma KB and human liver carcinoma Hepa59T/VGH cells [7,8]. Moreover, it has been found to control the proliferation of oral cancer Ca9-22 cells by inducing apoptosis, oxidative stress and DNA damage [9]. 5-*epi*-sinuleptolide (5-*epi*-SNEP) stimulated apoptosis of skin cancer cells through death receptor- and mitochondria-mediated caspase pathways [10]; furthermore, 5-*epi*-SNEP acetate induced reactive oxygen species (ROS) generation, calcium accumulation and matrix metallo proteinase (MMP) disruption, as well as apoptosis in human promyelocytic leukemia cells [11]. Finally, several norcembranoids isolated from the Indian soft coral *Sinularia kavarattiensis* were found to possess an inhibitory activity against the replication of *Chikungunya* virus (CHIKV) [12]. On the basis of this wide range of biological activities, we applied a combination of mass spectrometry (MS)-based proteomic approaches for target identification of 5-*epi*-SNEP. In particular, our strategy was based on the coupling of conventional affinity chromatography of immobilized small molecules, which obtains the specific interactors from a cell lysate (Figure 1A), with the drug affinity responsive target stability (DARTS) approach, which avoids chemical modification of the natural product [13,14,15,16]. DARTS is based on the evidence that a protein is less susceptible to proteolysis when bound to a small molecule. On this basis, once the small molecule has been exposed to the cell lysate, the treatment of the sample with a protease will give information on the potential specific targets of the small molecule its-self (Figure 1B). Since matrix-based proteomic methods suffer of limitations, in that immobilization and affinity purification could affect the activity of the small molecule, DARTS is an ideal complement for confirming the chemical proteomics results, also providing evidence for a direct interaction between the counterparts. In our study, both approaches pointed to the identification of actin proteins as main 5-*epi*-SNEP cellular targets. Finally, a biological investigation on 5-*epi*-SNEP effects on the microtubule cytoskeleton assembly was performed.

## 2. Results

Our combined proteomic approaches can be summarized in the following steps: (1) generation of a functional 5-*epi*-SNEP affinity matrix, (2) identification of 5-*epi*-SNEP interactome by affinity chromatography coupled with bioinformatics and western blotting, (3) application of DARTS on 5-*epi*-SNEP, (4) in-cell biological evaluation of 5-*epi*-SNEP activity.

### 2.1. Generation of a Functional 5-epi-SNEP Affinity Matrix

**Figure 2:**
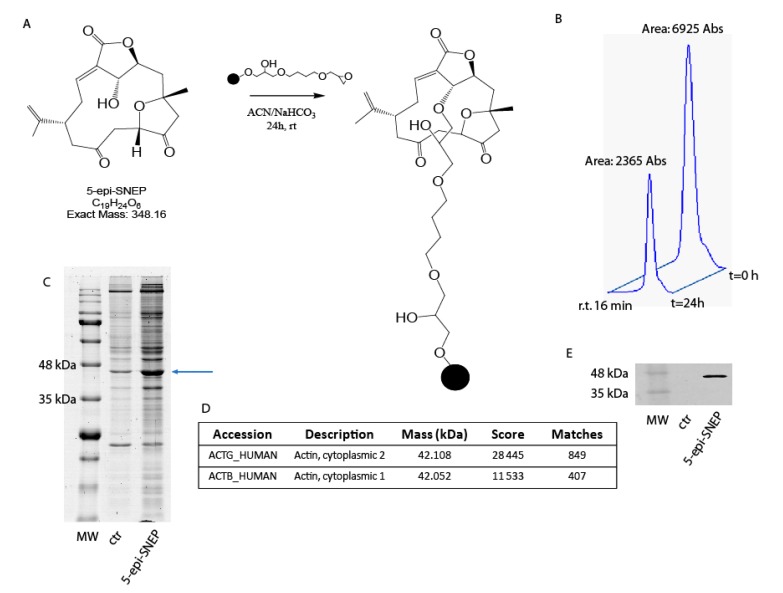
(**A**) Chemical structure of 5-*epi*-SNEP and its reaction with the epoxy-resin; (**B**) HPLC profile of free 5-*epi*-SNEP before and after coupling reaction; (**C**) SDS-PAGE of the eluted proteins from 5-*epi*-SNEP-bearing and control-beads; the arrow indicates the actin proteins; (**D**) Most abundant partners fished out by 5-*epi*-SNEP, reported together with the Mascot protein score and the peptide matches obtained by Mascot software; (**E**) Western blot analysis on proteins eluted from control and 5-*epi*-SNEP-beads using an antibody against actin isoforms.

On the basis of 5-*epi*-SNEP structural features, we selected Sepharose 6B beads bearing a highly reactive epoxy group as the best matrix to achieve the step of immobilization. The connection was easily accomplished through reaction of the hydroxyl function at C-5 on 5-*epi*-SNEP and the epoxy groups on the matrix (Figure 2A). Reaction yield was assessed at about 66% by reverse phase-high pressure liquid chromatography (RP-HPLC), comparing the peak area of 5-*epi*-SNEP at 220 nm in solution at the beginning of the reaction and after 24 h (Figure 2B). The remaining un-reacted epoxy groups on the resin were then quenched with a mixture water: isopropanol; an aliquot of Sepharose 6B, solely treated with the same solution, was used as resin control sample.

### 2.2. Identification of 5-epi-SNEPinteractome by Nano-LC-MSMS Analysis and Western Blotting

5-*epi*-SNEP-bearing and control beads were separately incubated with samples of HeLa cell protein extracts for 24 h at 4 °C, to promote interaction between the immobilized compound and its potential partner(s). The matrix beads were then isolated by centrifugation and washed with phosphate buffer (PBS) to reduce the amount of aspecific ligands weakly bound to the solid matrix, while the tightly bound proteins, most likely specifically fished out by 5-*epi*-SNEP, were released using a highly denaturant buffer compatible with the subsequent 1D-SDS-PAGE analysis. The protein mixtures eluted from 5-*epi*-SNEP-bearing and control beads were resolved by SDS-PAGE (Figure 2C), and the corresponding entire gel lanes were divided into several pieces and subjected to an in situ trypsin digestion protocol [17]. The tryptic peptide mixtures coming from each gel slice were then analyzed through nano-flow RP-HPLC MS/MS, and the MS/MS data, once converted into peak lists, were submitted Mascot for protein identification to Mascot software analysis. The list of the 5-*epi*-SNEP potential interactors was refined by superimposition of two independent experiments, and removing the proteins shared with the control experiments, giving a more restricted and confident list of putative specific 5-*epi*-SNEP interactors (Table S1). Actins have been recognized as main partners of 5-*epi*-SNEP, as is clearly evident by the high Mascot score reported in the identification process (Figure 2D). 

We then aimed to validate the method confirming the interactions of 5-*epi*-SNEP with actin by immuno-blotting analysis, as reported in Figure 2E. A significant antigen/antibody reaction was monitored in the 5-*epi*-SNEP gel lane compared to the control, providing a clear indication of a specific enrichment of the actin proteins due to the interaction with the marine compound.

### 2.3. Application of DARTS Protocol on 5-epi-SNEP Target Protein

**Figure 3:**
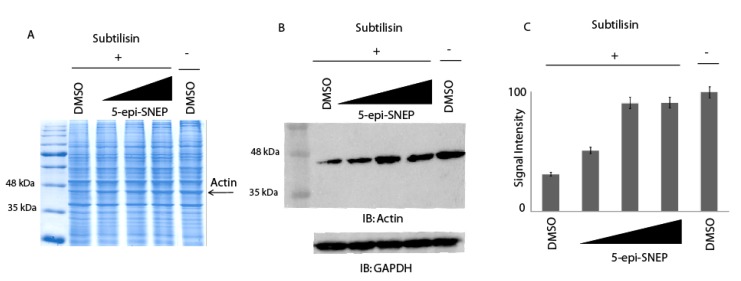
Enrichment of actin protection to protease upon 5-*epi*-SNEP interaction: DARTS using HeLa cell lysates treated or not with increasing quantities of 5-*epi*-SNEP from 20 ng to 2 μg; samples were subjected to subtilisin digestion and detected by Comassie staining (**A**) and by immunoblotting using an anti-actin antibody. GAPDH was resistant to subtilisin under these conditions and served as a loading control (**B**). (**C**) Densitometric analysis of immunoblotting assay using an anti-actin antibody reveals a high degree of protection versus protease action upon 5-*epi*-SNEP putative binding. Histogram was the result of image quantification analysis of two independent experiments, setting the intensity of undigested actin as 100%.

For a further validation of the above results, different quantities of native 5-*epi*-SNEP were incubated with HeLa cell lysates and the samples were then submitted to limited proteolysis with subtilisin. Two samples of cell lysates were treated with dimethyl sulfoxide (DMSO), and only one with subtilisin, representing the control experiments. All the samples were then submitted to 1D-SDS-PAGE and alternately either colored by Coomassie staining, or subjected to an immunoblotting analysis using an antibody specific for actin. As previously described, since the drug binding protects the proteins from the proteolysis, either globally or locally, it is thus possible to monitor the protease sensitivity of a particular target protein through a simple visualization by Coomassie staining (using HRMS analysis to identify the protein into gel bands as reported in Table S2) or by a semi-quantitative analysis by immunoblotting. As shown in Figure 3, increasing concentrations of 5-*epi*-SNEP undoubtedly protects actin from the enzyme proteolytic action when compared to the control samples exposed or not to subtilisin (first and last lane of the gel, respectively). This evidence also confirms the direct interaction of the marine natural compound with its target protein. 

### 2.4. Biological Evaluation of 5-epi-SNEP Action on Actin by in-Cell Assays

**Figure 4:**
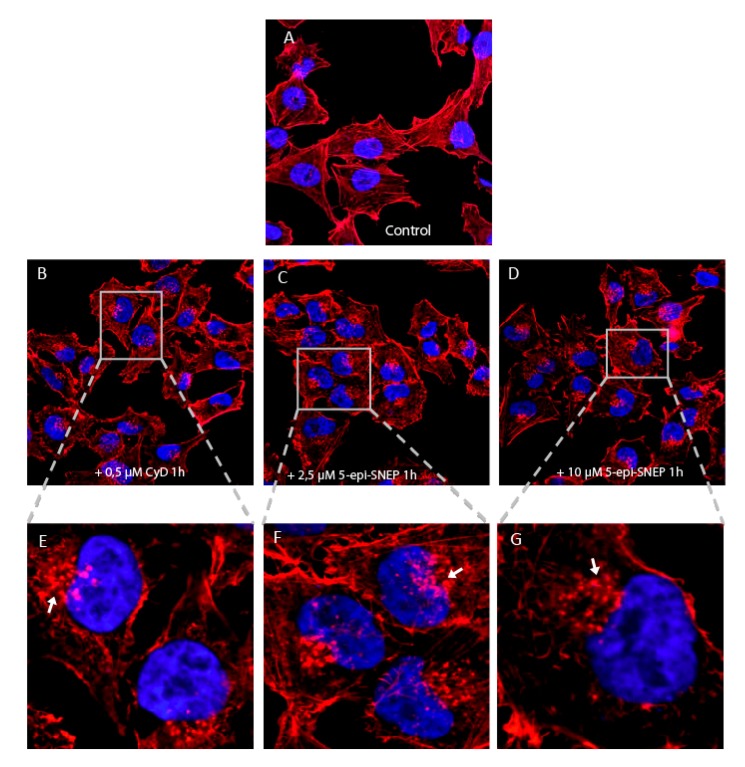
(**A**–**G**) Effect of 5-*epi*-SNEP on F-actin. HeLa cells were treated with vehicle control (**A**), or with two different concentrations (2.5 and 10 μM) of 5-*epi*-SNEP (**C**,**D**), for 1 h. CyD (**B**) at 0.5 μM for 1 h was used as positive control for actin filament depolymerization. Actin was labeled with TRITC-phalloidin and acquired by confocal microscope. Cells treated with CyD (**B**) show large actin aggregates (**E**, enlarged image, white arrow), as expected. A similar phenotype was observed in cells treated with 5-*epi*-SNEP at both concentrations (**C**,**D**) as shown in enlarge images (**F**–**G**, white arrow). (63× magnification. Red: F-actin; blue: nuclei).

Intrigued by the promising results obtained by proteomics, we examined the effects of 5-*epi*-SNEP on HeLa cell viability and on the cytoskeleton. As reported in Figure S2, neither 5-*epi*-SNEP nor its derivatives with a short epoxide-alkyl chain affected cell viability up to 50 μM. Thus, we chose 5-*epi*-SNEP concentrations of 2.5 and 10 μM to study its effect on the cytoskeleton, incubating HeLa living cells with the natural molecule for 1 h. Fluorescence micrographs of the actin cytoskeletons, stained with tetramethylrhodamine (TRITC)-phalloidin, were obtained by scanning confocal microscopy. As reported in Figure 4C–G, 5-*epi*-SNEP already induced a partial disruption of the actin cytoskeleton, compared to the control (Figure 4A), at the lowest concentration, as evidenced by the decrease of actin fibers and the formation of F-actin amorphous aggregates (arrows in Figure 4). At all concentrations, small actin aggregates co-existed with actin microfilament bundles and diffuse cortical F-actin (Figure 4C–G). Cytochalasin D (Figure 4B, CyD), used as a positive control, showed a similar behavior for actin filament depolymerization [18]. No significant effects on cell cycle were observed in neither 5-epi-SNEP treated cells nor in CyD treated ones (Figure S3).

## 3. Discussion and Conclusions

Target discovery in the area of bioactive natural compounds is a key aspect for understanding their biological effects. Here, the cellular interactome of the marine norditerpene 5-*epi*-sinuleptolide, isolated from the soft coral genus *Sinularia* and endowed with remarkable biological activities, has been analyzed coupling chemical proteomics and DARTS approaches. Both strategies indicated actin(s) to be the main target(s) of the marine natural compound. Actins have been identified by an extremely high Mascot score in the proteomic experiments, suggesting a strong affinity between 5-*epi*-SNEP and this protein, as confirmed by immunoblotting analysis. Furthermore, as reported by DARTS analysis, actin becomes less susceptible to proteolysis during the treatment of the cell lysates with increasing concentrations of 5-*epi*-sinuleptolide, validating the chemical proteomics results and proving the direct interaction between the counterparts. Finally, the following biological assays gave evidence of a strong perturbation of actin cytoskeleton, with the formation of actin aggregates induced by marine norditerpene without affecting cell viability, even at high concentration. 

Nowadays, alterations in the cytoskeleton structure and dynamics are considered a remarkable symptom of several diseases, such as cancer [19] and neurodegenerative disorders [20]. Cytoskeleton disassembly usually leads to protein abnormal aggregates, called inclusion bodies (IBs)—which in turn lead to protein misfolding [21]. Actually, one of the most efficient manners of perturbing actin involves the use of natural actin toxins, such as the lactone macrolides cytochalasins and latrunculins, which significantly depolymerize F-actin, inducing the formation of aggregates. Both compounds are widely used to test the contribution of actin in cellular events, such as endo/exocytosis, cell motility and migration, cell polarity. Since these compounds have no specificity for different types of actin, such as cardiac, smooth muscle, muscle and cytoskeletal form, their use as potential drugs is impaired due to undesirable off-target effects. However, the actin-targeting molecules are suitable tools for shedding light on this complex part of the cells internal machinery [21]. Here, we propose 5-*epi*-sinuleptolide as a natural compound with a novel chemical structure capable of interfering with actin dynamics polymerization, and this could have a great impact on the study of cytoskeleton alterations at a molecular level.

## 4. Materials and Methods 

### 4.1. Generation of a Functional 5-epi-SNEP Affinity Matrix

Epoxy-activated Sepharose™ 6B matrix was swollen with water (200 μL/mg) for 60 min and extensively washed with water. 5-*epi*-SNEP (1 μmol) was diluted in 150 μL of 50% CH_3_CN/50% 100 mM sodium bicarbonate (NaHCO_3_), 1% (*v*/*v*) triethylammine and added to 100 μL of matrix at room temperature for 24 h with continuous shaking. A control matrix was obtained, under the same experimental conditions, without the metabolite. The amount of immobilized 5-*epi*-SNEP was estimated by integrating the peaks of the free 5-*epi*-SNEP after HPLC injections of supernatants at *t* = 0 and 24 h in a 1100 Series Chromatographer (Agilent, Santa Clara, CA, USA) equipped with a UV detector set at 220 nm. HPLC runs were carried out on a C18 column (Luna Omega 5 µm Polar C18 150 × 2.1 mm, Phenomenex, Torrance, CA, USA) at a flow rate of 0.200 mL/min. Elution was achieved by means of a linear gradient of B from 10% to 95% over 20 min (solution A: H_2_O and trifluoroacetic acid 0.1%; solution B: 95% CH_3_CN, 5% H_2_O and trifluoroacetic acid 0.07%). Both resins were washed 2 times with 300 μL of a mixture of water/isopropanol (1:2) and then incubated with 150 µL of the same solution for 3 h at room temperature and under continuous shaking to inactivate the free epoxy groups. Then, matrices were washed extensively with phosphate saline buffer (PBS) to remove traces of isopropanol. HeLa cells were grown in Dulbecco’s modified Eagle medium supplemented with 10% (*v*/*v*) fetal bovine serum, 100 U/mL penicillin, 100 mg/mL streptomycin, at 37 °C in a 5% CO_2_ atmosphere (all reagents were from Sigma–Aldrich, Darmstadt, Germany). Cells were collected by centrifugation (1000× *g*, 5 min), washed three times with PBS and resuspended in 1× ice cooled PBS containing Igepal (0.1%), supplemented with a protease inhibitor cocktail. The obtained suspensions were mechanically lysed at 4 °C using a Dounce homogenizer, and cellular debris were removed by centrifugation at 10,000× *g* for 5 min at 4 °C. Protein concentration was determined using Bradford assay (Bio-Rad) and adjusted to 3 mg/mL. 5-*epi*-SNEP–bound beads suspension (100 μL) and the same amount of the control unbound matrix were separately incubated with 1 mg of HeLa extracts under continuous shaking (24 h, 4 °C). The beads were collected and washed three times with PBS (pH 7.4). The bound proteins were eluted by boiling the beads in SDS-PAGE sample buffer (60 mM Tris/HCl pH 6.8, 2% SDS, 0.001% bromophenol blue, 10% glycerol, 2% 2-mercaptoethanol). The eluted proteins were separated on SDS-PAGE at 12% of acrylamide and stained with Coomassie G-250 (Bio-Rad, Hercules, CA, USA). The experiment was repeated twice.

### 4.2. Identification of 5-epi-SNEP Interactome by Nano-LC-MSMS Analysis and Western Blotting

The entire SDS-PAGE gel lanes relative to the 5-*epi*-SNEP-based and control experiments were cut into 10 pieces and digested. Each piece was washed with ultrapure water and CH_3_CN and subjected to in situ protein digestion as described by Shevchenko [17]. Briefly, each slice was reduced with 6.5 mM 1,4-dithiothreitol and alkylated with 54 mM iodoacetamide, then washed and rehydrated in trypsin solution (12 ng/μL) on ice for 1 h. After the addition of ammonium bicarbonate (40 μL, 50 mM, pH 8.5), protein digestion was allowed to proceed overnight at 37 °C. The supernatant was collected and peptides were extracted from the slices using 100% CH_3_CN, and both supernatants were combined. The peptide samples were dried and dissolved in formic acid (FA, 10%) before MS analysis. The peptide mixture (5 μL) was injected into a nano-ACQUITY UPLC system (Waters, Milford, MA, USA). Peptides were separated on a 1.7-μm BEH C18 column (Waters) at a flow rate of 280 nL/min. Peptide elution was achieved with a linear gradient (solution A: 95% H_2_O, 5% CH_3_CN, 0.1% acetic acid; solution B: 95% CH_3_CN, 5% H_2_O, 0.1% acetic acid); using a linear gradient of B from 20% to 90% over 53 min. MS and MS/MS data were acquired on an Orbitrap LTQ XL high-performance liquid chromatography MS system (Thermo-Scientific, Waltham, MA, USA ) equipped with an electrospray source (ESI). The five most intense doubly and triply charged peptide ions were chosen and fragmented. The resulting MS data were processed by MS Converter General User Interface software (ProteoWizard; http://proteowizard.sourceforge.net/project.shtml) to generate peak lists for protein identifications. Database searches were carried out on the Mascot Deamon version 4.1 by Matrix Science (London, UK). The SwissProt database (release January 2017, 553474 sequences, 198069095 residues) was employed (settings: two missed cleavages; carbamidomethyl (C) as fixed modification and oxidation (M) and phosphorylation (ST) as variable modifications; peptide tolerance 80 ppm; MS/MS tolerance 0.8 Da).

5-*epi*-SNEP-modified bead suspension (50 μL) and the same amount of the unmodified matrix were treated as described above and the eluates were analyzed by Western blotting. Briefly, each sample was resolved on a 12% SDS-PAGE gel and transferred onto a nitrocellulose membrane. The membrane was incubated for 1 h in a blocking solution containing 30 Mm Tris pH 8, 170 mM NaCl, 3.35 mM KCl, 0.05% Tween-20, 5% non-fat dried milk, then incubated overnight at 4 °C with primary monoclonal antibody raised against actin (1:500, Santa Cruz Biotechnology, Inc., Dallas, TX, USA). Membranes were then incubated for 1 h with mouse peroxidase-conjugated secondary antibody (1:2500; Thermo-Scientific). The signal was detected using an enhanced chemiluminescent substrate and LAS 4000 (GE Healthcare, Waukesha, WI, USA) digital imaging system. The experiment was repeated twice.

### 4.3. Optimization of DARST Protocol on 5-epi-SNEP Target Protein

Hela cells were lysed as reported above and supplemented with protease inhibitors. Protein concentration was determined by Bradford assay (Bio-Rad). Lysates (300 μg in 100 μL) were incubated with DMSO control, or 5-*epi*-SNEP from 0.2 ng/μL to 20 ng/μL, for 1 h at room temperature under continuous shaking. Then, samples underwent proteolysis with subtilisin (subtilisin to protein ratio of 1:1000 for 30 min at 25 °C with continuous shaking), were boiled in SDS-PAGE sample buffer (60 mM Tris/HCl pH 6.8, 2% SDS, 0.001% bromophenol blue, 10% glycerol, 2% 2-mercaptoethanol) to stop the digestion, and loaded on a 12% SDS-PAGE followed by Comassie staining or Western blot analysis using an anti-actin antibody (1:500, Santa Cruz Biotechnology, Inc.). Membranes were then incubated for 1 h with mouse peroxidase-conjugated secondary antibody (1:2500; Thermo-Scientific). The experiment was repeated twice.

The signal was detected using an enhanced chemiluminescent substrate and LAS 4000 (GE Healthcare, Waukesha, WI, USA) digital imaging system. 

The entire SDS-PAGE gel lanes relative to the 5-*epi*-SNEP-based and control experiments were cut into 10 pieces and digested as reported above.

### 4.4. Biological Evaluation of 5-epi-SNEP Action by in-Cell Assays

The human uterine cervical cancer cells HeLa were obtained from the American Type Culture Collection (ATCC), and were cultured in DMEM medium (Euroclone, Milan, Italy) supplemented with 10% (*v*/*v*) fetal bovine serum (Euroclone), 100 U/mL penicillin and 100 µg/µL streptomycin (Euroclone) at 37 °C in a 5% CO_2_ atmosphere. 

To determine the effect of 5-*epi*-SNEP on cell viability, we performed the colorimetric MTT metabolic activity assay. 5 × 10^4^ HeLa cells were cultured in a 96-well plate and, after 24 h, exposed to multiple concentrations of 5-*epi*-SNEP (100, 50, 25, 10, 5, 2.5 and 1 µM) for 24 h. Then, 10 µL of MTT (5 mg/mL) was added in each well. After 1 h at 37 °C, the supernatants were removed and the resultant formazan crystals were dissolved in 100 µL DMSO. The absorbance intensity was measured by a microplate reader at 550 nm with a reference wavelength of 620 nm. All experiments were performed in quadruplicate, and the relative cell viability (%) was expressed as a percentage relative to the untreated control cells. 

The cell cycle analysis was performed on the HeLa cell line. To synchronize the cells, HeLa was seeded in 24 multiwell-plate in growth medium with 10% FBS overnight. Then, the cells were rinsed by PBS and changed to serum free medium for 24 h. After serum starvation, the cells were treated with the reported concentrations of 5-*epi*-SNEP and CyD for 24 h in the presence of serum to release them into cell cycle. For FACS analysis, cell samples were harvested by trypsinization and stained with propidium iodide (20 µg/mL) for 30 min at 4 °C in the dark. The cells were analyzed using the FACScan cytometer (BD Bioscience, San Jose, CA), and the ModFit LT software program version 3.2 (Verity Software House, Inc., Maine, CA, USA) was used to determine the distribution of cells in each phase of the cell cycle (G_1_, S, and G_2_).

In order to analyze the effect of 5-*epi*-SNEP on F-actin, 4 × 10^4^ HeLa cells per well were seeded on cover slips in 24-well plastic plates. After 24 h, cells were treated or not with 5-*epi*-SNEP (2.5 or 10 µM) for 1 h. Cytochalasin D (CyD) treatment (0.5 µM for 1 h) was used as positive control for actin filament depolymerization. Then, cells were fixed with 4% paraformaldehyde in PBS for 10 min, permeabilized and blocked in 1% bovine serum albumine for 30 min. Actin microfilaments and nuclei were stained, respectively, with 2 µg/mL TRITC-phalloidin Sigma-Aldrich, (Darmstad, Germany), Thermo-Scientific (Waltham, MA, USA) for 1 h. The images were collected on a Zeiss LSM 510 confocal microscope (Carl Zeiss Microscopy GmbH, Oberkochen, Germany) using LSM 510 Meta software 4.0 SP2 version. The images shown are representative of multiple fields and triplicate cover slips per experiment.

## Supplementary Materials

The following are available online at www.mdpi.com/1660-3397/15/10/312/s1, Figure S1: Scheme of 5-*epi*-SNEP modification by glycidol and tandem MS analysis of the reaction products, Figure S2: Cell viability of HeLa cells treated by 5-*epi*-SNEP or by its derivatives, Figure S3: Analysis of HeLa cell cycle in presence and in absence of 5-*epi*-SNEP and Cytochalasin D, Table S1: List of 5-*epi*-SNEP partners found in HeLa cell lysate, Table S2: Actin identification by tandem MS experiments in three samples of HeLa cell lysate treated or not with 5-*epi*-SNEP and subtilisin.
