# Supplementary material for: Discovering the Biological Target of 5-epi-Sinuleptolide Using a Combination of Proteomic Approaches
Source: Mar Drugs. 2017 Oct 13;15(10):312. doi: 10.3390/md15100312 (PMC5666420; doi:10.3390/md15100312)
Supplement: Supplementary file 1 [file marinedrugs-15-00312-s001.pdf]

Discovering the biological target of 5-epi-Sinuleptolide using a combination of proteomic approaches.

Elva Morretta[1,2], Roberta Esposito[1], Carmen Festa[3], Raffaele Riccio[1],* Agostino Casapullo[1] and Maria Chiara Monti[1,*]

[1] Department of Pharmacy, University of Salerno, Via Giovanni Paolo II 132, 84084, Fisciano, Salerno, Italy; emorretta@unisa.it, roesposito@unisa.it, riccio@unisa.it,casapullo@unisa.it,mcmonti@unisa.it

[2] PhD Program in Drug Discovery and Development; University of Salerno, Via Giovanni Paolo II 132, 84084, Fisciano, Salerno, Italy;

[3] Department of Pharmacy, University of Naples "Federico II", Naples, Italy; carmen.festa@unina.it

* Correspondence: riccio@unisa.it, mcmonti@unisa.it; tel.: 0039-089-969754

Chemical modification of 5-epi-SNEP by glycidol and evaluation of its biological activity:

Generally when a molecule is functionalized for a pull-down experiment, it's valuable to verify if the chemical modification impacts on compound activity. Thus, we moved to check if 5-epi-SNEP modification by an epoxide containing molecule, such as glycidol, affected its cellular toxicity versus HeLa cells. To this aim, 1 mg of 5-epi-SNEP was incubated with glycidol (Figure S1A, glycidol excess of 250 X over 5-epi-SNEP (w:w)) for 1 h at 37°C in ACN-50 mM $NaHCO_3$ 1% triethyl-amine and the products were purified by HPLC on a C18 column (Luna Omega 5µm Polar C18 150× 2.1mm, Phenomenex, Torrance, CA) at a flow rate of 0.2 ml/min. Elution was achieved by means of a linear gradient of B from 10 to 95% over 20 min (solution A: H_2O and trifluoroacetic acid 0.1%; solution B: 95% CH_3CN, 5% H_2O and trifluoroacetic acid 0.07%). The compound mixture **1A** and **1B** in their mono-sodiated forms (Figure S1A) has been characterized by HRMS and HRMSMS analysis on an Orbitrap LTQ XL (Thermo-Scientific) giving a single m/z ion at 427.1722 u.m.a (chemical formula in Fig. S1B). As shown in Figure S1B, compound **1** was not detected by HRMS analysis since it easily dehydrated giving a mixture of products, well characterized by HR-MSMS analysis. Indeed, as reported in Figure S1C, there are fragments compatible with: the loss of one molecule of H_2O from compound 1B (m/z 409.1755 u.m.a) or the loss of one molecule of CO from compound 1A (m/z 399.1773 u.m.a) pointing that only compounds 1A and 1B were formed. Moreover, the m/z fragments at 371.1462 u.m.a and 353.1357 u.m.a, compatible with the loss of the glycidol-derived side chain, confirmed the mechanism postulated in Figure S1A. The obtained mixture was submitted to MTT assays (as reported in paragraph 4.4) and the results are showed in Fig.S2. The histograms clearly demonstrated that there is no HeLa cells cito-toxicity alteration if compared with the native unaltered 5-epi-SNEP molecule.

Scheme of 5-epi-SNEP modification by glycidol and tandem MS analysis of the reaction products.

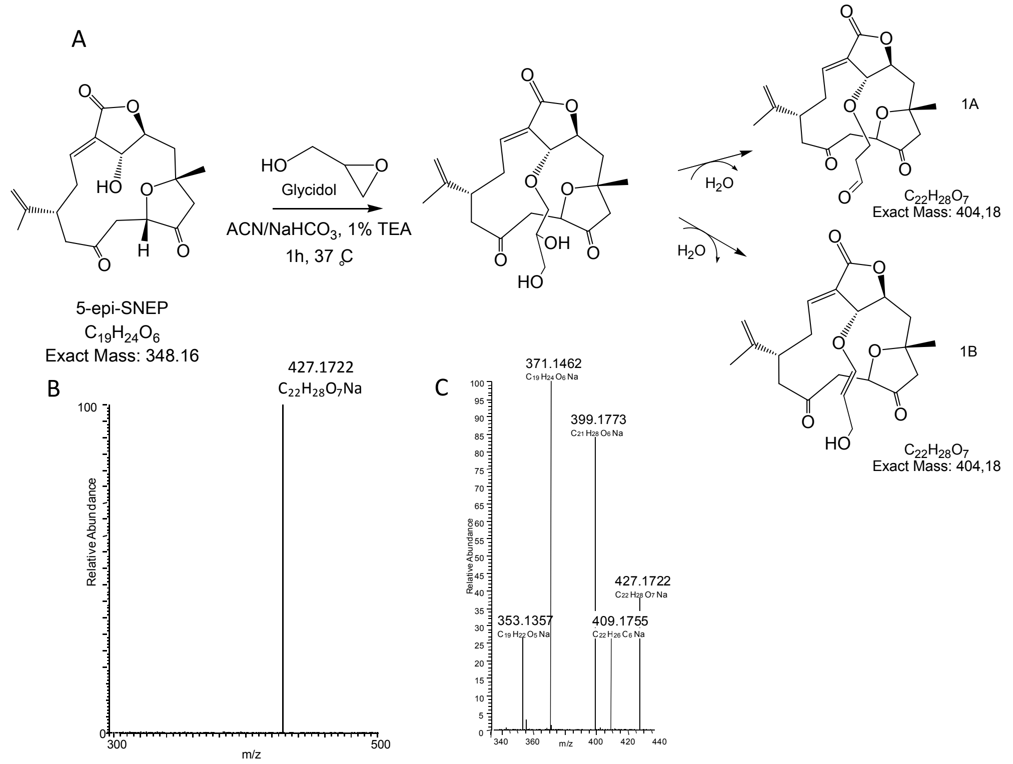

Figure S1: Panel A shows the postulated mechanism of 5-epi-SNEP modification by glycidol followed by dehydration. Panel B and C show the HRMS analysis of the reaction products and their HRMSMS spectra together with the chemical formulas.

Cell viability of HeLa cells treated by 5-epi-SNEP or by its derivatives.

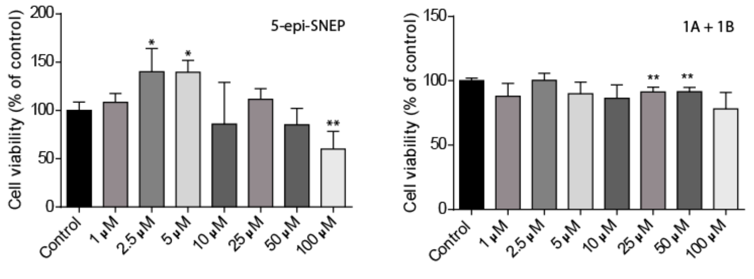

Figure S2: Cell viability assay of HeLa cells treated with 5-epi-SNEP (left panel) or compounds mixture 1A and 1B (right panel) for 24 h at reported concentrations. Histograms were the result of four independent experiments and the cell viability of control was put as 100%.

List of 5-epi-SNEP partners found in HeLa cell lysate.

Table S1: For each protein, the following parameters are reported: Mascot score (Score), molecular weight (mass), number of matched peptides (Matches) and unique peptides used in the identification process (Match(sig)), number of sequences (Sequences) and the number of significant distinct sequence matches in the protein identification process (Seq(sig). All the proteins with a Mascot Score less than 60 and identified with less than 5 Match(sig) were removed.

Accession	Score	Mass	Matches	Match(sig)	Sequences	Seq(sig)	Description
ACTG_HUMAN	28445	42.108	849	743	22	20	Actin, cytoplasmic 2
ACTB_HUMAN	11533	42.052	407	291	10	6	Actin, cytoplasmic 1
ACTC_HUMAN	3682	42.334	323	136	7	4	Actin, alpha cardiac muscle 1
ACTBL_HUMAN	1697	42.318	104	67	6	5	Beta-actin-like protein 2
TBB4B_HUMAN	1623	50.255	73	63	17	15	Tubulin beta-4B chain
TBA1C_HUMAN	1118	50.548	53	39	10	10	Tubulin alpha-1C chain
1433E_HUMAN	1064	29.326	29	26	8	6	14-3-3 protein epsilon
ANXA1_HUMAN	996	38.918	28	26	9	8	Annexin A1
VDAC1_HUMAN	768	30.868	27	23	8	6	Voltage-dependent anion-selective channel protein 1
LDHB_HUMAN	693	36.900	32	28	5	3	L-lactate dehydrogenase B chain
EF2_HUMAN	685	96.246	37	25	16	10	Elongation factor 2
CAZA1_HUMAN	604	33.073	28	25	5	4	F-actin-capping protein subunit alpha-1
1433Z_HUMAN	518	27.899	22	21	5	5	14-3-3 protein zeta/delta
TERA_HUMAN	456	89.950	22	16	10	7	Transitional endoplasmic reticulum ATPase
ARF1_HUMAN	431	20.741	11	9	5	4	ADP-ribosylation factor 1
TPM3_HUMAN	405	32.987	28	17	6	5	Tropomyosin alpha-3 chain
PSA7_HUMAN	302	28.041	7	6	4	3	Proteasome subunit alpha type-7
COF2_HUMAN	297	18.839	19	13	3	3	Cofilin-2
PGAM1_HUMAN	287	28.900	20	10	5	4	Phosphoglycerate mutase 1
RAN_HUMAN	273	24.579	9	8	2	2	GTP-binding nuclear protein Ran
ARF4_HUMAN	255	20.612	6	5	3	2	ADP-ribosylation factor 4
VDAC3_HUMAN	244	30.981	44	18	5	4	Voltage-dependent anion-selective channel protein 3
ADT3_HUMAN	242	33.073	13	12	3	3	ADP/ATP translocase 3
TBB6_HUMAN	226	50.281	12	9	6	4	Tubulin beta-6 chain
VDAC2_HUMAN	220	32.060	10	7	3	3	Voltage-dependent anion-selective channel protein 2
PRDX4_HUMAN	213	30.749	12	10	3	3	Peroxiredoxin-4
PPIB_HUMAN	180	23.785	16	8	4	3	Peptidyl-prolyl cis-trans isomerase B
ANXA5_HUMAN	177	35.971	17	13	7	5	Annexin A5
1433T_HUMAN	175	28.032	9	8	3	3	14-3-3 protein theta
IF4A1_HUMAN	174	46.353	8	7	4	4	Eukaryotic initiation factor 4A-I
DEST_HUMAN	162	18.950	9	6	3	2	Destrin
PRDX6_HUMAN	145	25.133	8	5	5	4	Peroxiredoxin-6
PCNA_HUMAN	145	29.092	9	5	5	3	Proliferating cell nuclear antigen
RS10_HUMAN	116	18.886	13	10	3	2	40S ribosomal protein S10
RS18_HUMAN	98	17.708	6	5	2	2	40S ribosomal protein S18
APT_HUMAN	90	19.766	7	6	2	2	Adenine phosphoribosyltransferase
RANG_HUMAN	86	23.467	5	5	3	3	Ran-specific GTPase-activating protein

Actin identification by tandem MS experiments in three samples of HeLa cell lysate treated or not with 5-epi-SNEP and subtilisin

Table S2: The following parameters are reported for each identification: Mascot score (Score), number of matched peptides (Matches) and the relative quantization of actin (emPAI). The so-called exponentially modified protein abundance index (emPAI) was based on the number of observed peptides per protein normalized by the theoretical number of protein theoretical peptides. [1] If 1 mg of HeLa cell lysate is not treated with 5-epi-SNEP and it is digested by subtilisin (DMSO+subtilisin), actin is identified with low score, few matches and low emPAI, clear indication of its susceptibility to proteases. When 1 mg of HeLa cell lysate is treated with 20 ng of 5-epi-SNEP and it is digested by subtilisin (5-epi-SNEP+subtilisin), actin is identified with higher score, many matches and high emPAI, as expected for a 5-epi-SNEP-mediated protection to protease action. The last column reports the identification of actin in absence of subtilisin and 5-epi-SNEP as it is in the HeLa cell lysate.

	DMSO + Subtilisin			5-epi-SNEP + Subtilisin			DMSO -Subtilisin		
Description	Score	Matches	emPAI	Score	Matches	emPAI	Score	Matches	emPAI
Actin, cytoplasmic 1 Homo sapiens	50	16	0.28	1986	117	4.11	4146	240	7.45

Analysis of HeLa cell cycle in presence and in absence of 5-epi-SNEP and Cytochalasin D

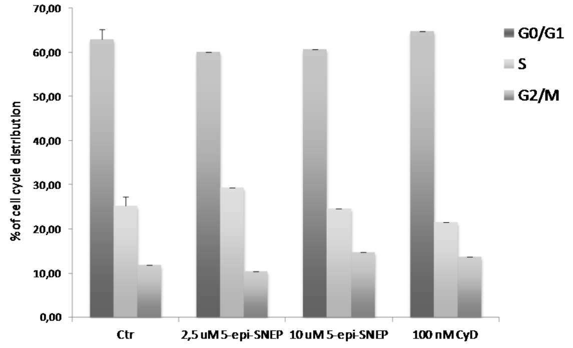

Figure S3: Cells were treated with the reported concentrations of 5-epi-SNEP and CyD for 24 hours. Cell cycle was evaluated by flow cytometry. The percentage of cell distribution in each phase was measured by ModFit LT software program (G_1, S, and G_2). Data are mean of four independent experiments ± S.D.

References:

1. Shinoda, K.; Tomita, M.; Ishihama, Y.; emPAI Calc—for the estimation of protein abundance from large-scale identification data by liquid chromatography-tandem mass spectrometry *Bioinformatics*, **2010,** 26, 576–577 doi:10.1093/bioinformatics/btp700.
